# Palaeogeographic implications of a new iocrinid crinoid (Disparida) from the Ordovician (Darriwillian) of Morocco

**DOI:** 10.7717/peerj.1450

**Published:** 2015-12-07

**Authors:** Samuel Zamora, Imran A. Rahman, William I. Ausich

**Affiliations:** 1 Instituto Geológico y Minero de España, Zaragoza, Spain; 2 School of Earth Sciences, University of Bristol, Bristol, United Kingdom; 3 School of Earth Sciences, Ohio State University, Columbus, OH, United States

**Keywords:** Crinoidea, Paleozoic, Micro-CT, Paleogeography, Morocco, Ordovician

## Abstract

Complete, articulated crinoids from the Ordovician peri-Gondwanan margin are rare. Here, we describe a new species, *Iocrinus africanus* sp. nov., from the Darriwilian-age Taddrist Formation of Morocco. The anatomy of this species was studied using a combination of traditional palaeontological methods and non-destructive X-ray micro-tomography (micro-CT). This revealed critical features of the column, distal arms, and aboral cup, which were hidden in the surrounding rock and would have been inaccessible without the application of micro-CT. *Iocrinus africanus* sp. nov. is characterized by the presence of seven to thirteen tertibrachials, three in-line bifurcations per ray, and an anal sac that is predominantly unplated or very lightly plated. *Iocrinus* is a common genus in North America (Laurentia) and has also been reported from the United Kingdom (Avalonia) and Oman (middle east Gondwana). Together with *Merocrinus*, it represents one of the few geographically widespread crinoids during the Ordovician and serves to demonstrate that faunal exchanges between Laurentia and Gondwana occurred at this time. This study highlights the advantages of using both conventional and cutting-edge techniques (such as micro-CT) to describe the morphology of new fossil specimens.

## Introduction

Ordovician crinoids from west peri-Gondwana (North Africa and southwestern and central Europe) are relatively rare, with only a few species reported from Spain, France, Italy, Morocco, Portugal, and the Czech Republic (Ubaghs, 1969; Ubaghs, 1983; Prokop & Petr, 1999; Ausich, Gil Cid & Domínguez Alonso, 2002; Ausich, Sá & Gutiérrez-Marco, 2007; Correia & Loureiro, 2009; Zamora, Colmenar & Ausich, 2014; Sumrall et al., 2015). Crinoids from Morocco include an incomplete specimen assigned to *Ramseyocrinus* sp. by Donovan & Savill (1988) from the Upper Fezouata Formation, which is Floian (Early Ordovician) in age (*sensu* Ausich, Sá & Gutiérrez-Marco, 2007), and several well-preserved complete specimens of *Rosfacrinus robustus* (Le Menn & Spjeldnaes, 1996), from the Upper Tiouririne Formation (Lefebvre et al., 2007), which is Katian (Late Ordovician).

Most of the crinoid genera from the Ordovician of peri-Gondwana are endemic, and this hampers our ability to understand the migration patterns of crinoids during this important time interval, in which several echinoderm classes reached major peaks in diversity (Guensburg & Sprinkle, 2000; Sprinkle & Guensburg, 2004; Nardin & Lefebvre, 2010; Lefebvre et al., 2013). Until now, the only exception was *Merocrinus*, which has been reported from England (Avalonia), Spain (peri-Gondwana), and North America (Laurentia) (Ausich, Gil Cid & Domínguez Alonso, 2002). Herein, we report a new species of *Iocrinus* from the Ordovician of Morocco, thereby extending the range of this genus with certainty to encompass west peri-Gondwana (in addition to Avalonia and Laurentia; Donovan et al., 2011) and confirming its cosmopolitan distribution. *Iocrinus africanus* sp. nov. is described based on a single well-preserved specimen, which was collected from south Alnif (eastern Anti-Atlas, Morocco) and is preserved in a concretion found in the Taddrist Formation, which is Darriwilian in age (Rábano, Gutiérrez-Marco & García-Bellido, 2014). The new crinoid was studied using both traditional techniques (casting the mould in latex) and X-ray micro-tomography (micro-CT). This allows us to describe the morphology of *Iocrinus africanus* sp. nov. in great detail and serves as a basis for comparison with other species of *Iocrinus*.

### Geological setting and stratigraphy

Ordovician outcrops are very well developed and exposed in the Anti-Atlas Mountains of Morocco (Destombes, Hollard & Willefert, 1985). Many units yield well-preserved specimens of echinoderms, a number of which are currently under study (e.g., Hunter et al., 2010; Van Roy et al., 2010; Van Roy, Briggs & Gaines, 2015; Sumrall & Zamora, 2011; Martin et al., in press), and these faunas occur throughout sections from the Lower to Upper Ordovician. Numerous clades of echinoderms have been documented, including stylophorans, solutes, blastozoans, crinoids, asteroids, edrioasteroids, and cyclocystoids.

**Figure 1:**
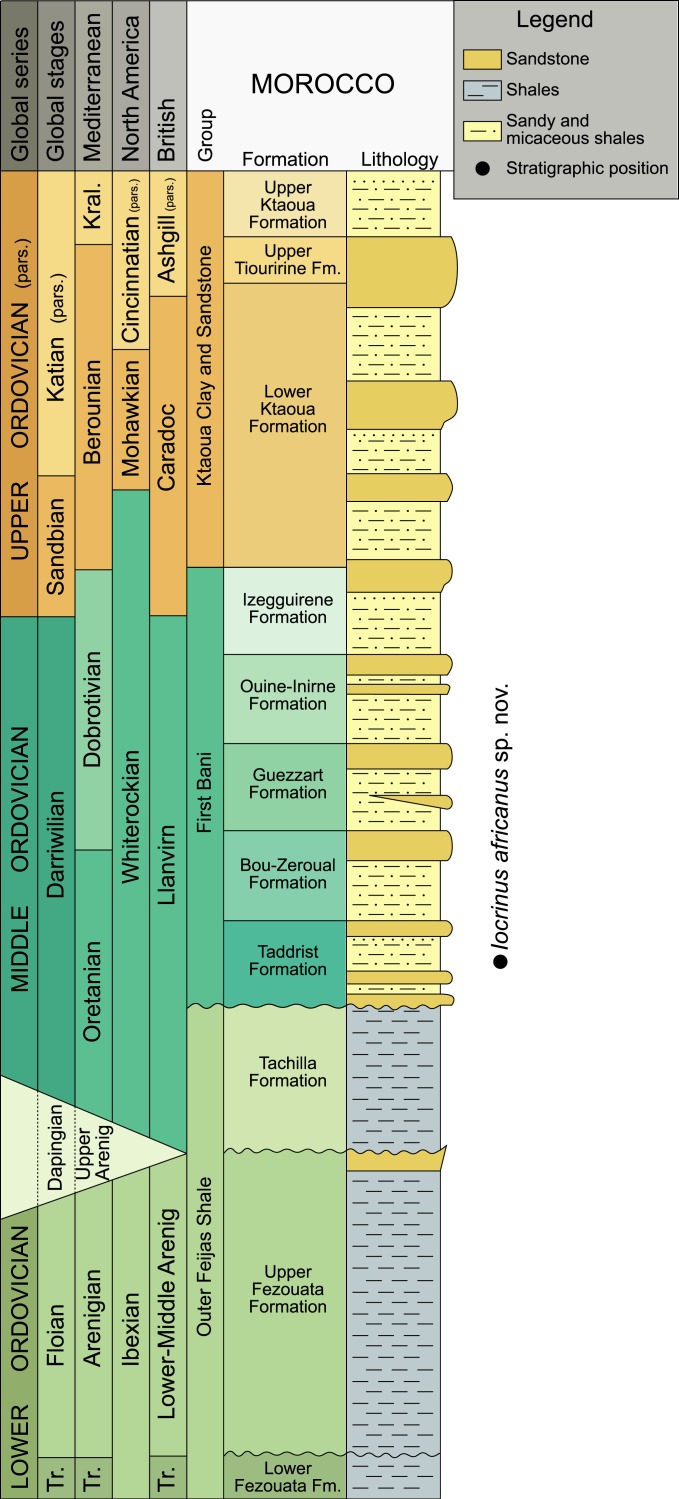
Chronostratigraphical chart for the Ordovician, indicating the levels that provided the studied specimen. Correlations between stratigraphical units in the Anti-Atlas (after Destombes, Hollard & Willefert, 1985; Gutiérrez-Marco et al., 2003; Villas et al., 2006), the British regional time scale (Fortey et al., 1995), North American graptolite zonal sequences (Webby et al., 2004), Mediterranean regional stages (Gutiérrez-Marco et al., 2003), and global stages are shown. Modified from Sumrall & Zamora (2011). Abbreviations: Kral, Kralodvorian; pars., partial; Tr., Tremadocian.

The Ordovician succession in the Anti-Atlas region is divided into the following lithostratigraphic units: the Outer Feijas Shale Group, the First Bani Sandstone Group, the Ktaoua Clay and Sandstone Group, and the Second Bani Sandstone Group (Choubert, 1943; Choubert & Termier, 1947; Destombes, Hollard & Willefert, 1985). The Outer Feijas Shale Group includes the Lower and Upper Fezouta formations (Tremadocian–Floian) and the Tachilla Formation (Darriwilian) (Fig. 1). These units are characterized by siltstones that are rich in graptolites, with some thin sandstone interbeds, and contain exceptionally preserved Burgess Shale-type faunas in places (Van Roy et al., 2010; Van Roy, Briggs & Gaines, 2015; Martin et al., in press). The overlying First Bani Group spans the Darriwilian to Sandbian and is subdivided into five formations (Taddrist, Bou-Zeroual, Guezzart, Ouine-Inirne, and Izegguirene formations) that are chiefly comprised of sandstones with interbedded shales. This group is the thickest, most constant, and most extensive sandstone group in the Anti-Atlas Mountains (Destombes, Hollard & Willefert, 1985). The fossil taxa recovered from the First Bani Group were reviewed by Gutiérrez-Marco et al. (2003), and there are no reports of crinoids from this time interval.

The First Bani Group is overlain by the Ktaoua Clay and Sandstone Group (Sandbian–Katian), which is comprised of siltstones interbedded with two or three sandstone units, depending on the exact position within the Anti-Atlas Mountains. It is divided into three units: the Sandbian to Katian Lower Ktaoua Formation, the Katian Upper Tiouririne Formation, and the Katian Upper Ktaoua Formation. The Ordovician ends with the Second Bani group, which is Hirnantian in age.

**Figure 2:**
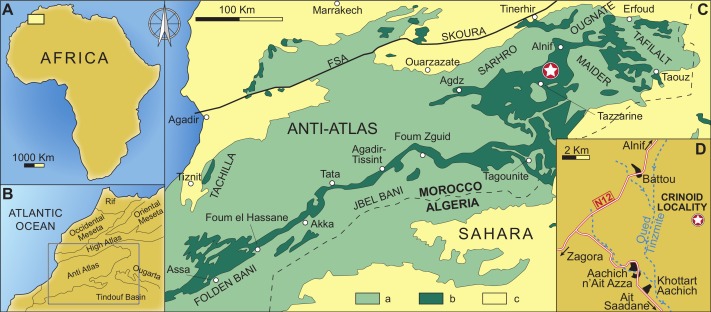
Geographical and geological setting of the eastern Anti-Atlas Mountains, Morocco, showing the type locality of the new species (indicated by a star) close to the village of Battou. After Rábano, Gutiérrez-Marco & García-Bellido (2014). (A) Map of Africa. (B) Detailed map of west Africa showing the position of the Anti-Atlas Mountains. (C) Simplified geological map of Morocco with the position of the studied locality; a: Precambrian and Palaeozoic rocks, b: Ordovician rocks, c: post-Palaeozoic cover. (D) Geographic map indicating the position of the studied locality.

**Figure 3:**
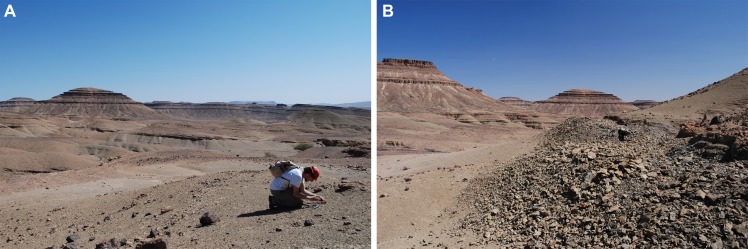
Field photographs showing the Taddrist Formation and the levels yielding fossiliferous concretions. (A) General view of the Taddrist Formation in the studied area. (B) Detail of the trench providing the fossiliferous concretions.

The new locality yielding *Iocrinus africanus* sp. nov. lies in the Taddrist Formation, low in the First Bani Group, close to the village of Battou (south Alnif, eastern Anti-Atlas) (Figs. 1 and 2). This locality was recently described by Rábano, Gutiérrez-Marco & García-Bellido (2014), who provided detailed information about the faunal content and age based on the presence of key graptolites and trilobites. In this area, the Taddrist Formation has been excavated predominantly by local collectors and has yielded a rich faunal assemblage preserved in carbonate concretions (Fig. 3). Rábano, Gutiérrez-Marco & García-Bellido (2014) suggested that the levels containing fossiliferous concretions belong to the *Didymograptus murchisoni* Biozone (Gutiérrez-Marco et al., 2003), which is assigned to the upper Oretanian, a regional stage roughly equivalent to the upper Darriwilian 2/basal Darriwilian 3 stage slices of the global chronostratigraphic scale (Gutiérrez-Marco, Sá & Rábano, 2008; Bergström et al., 2009). According to Rábano, Gutiérrez-Marco & García-Bellido (2014), the fossiliferous concretions have yielded the trilobites *Caudillaenus nicolasi* (Rábano, Gutiérrez-Marco & García-Bellido, 2014), *Morgatia*? *rochi* (Destombes, 1972), *Placoparia* (*Coplacoparia*) sp. nov., *Colpocoryphe* sp., *Parabarrandia* aff. *crassa* (Barrande, 1872), and an undetermined cheirurid (*Eccoptochile*? sp.). Other non-trilobite fossils include molluscs (e.g., a cyrtonellid tergomyan, bivalves such as *Praenucula* sp., and orthoconicnautiloids), hyoliths (*Elegantilites* sp.), echinoderms (Diploporita and Asterozoa indet.), conularids (*Exoconularia* sp.), and rare graptolites (*Didymograptus* sp.). In addition to the crinoid described herein, new cyclocystoids, the first ever reported from Africa, were recently presented from this locality and await formal description (Sprinkle, Reich & Lefebvre, 2015).

## Material and Methods

The studied specimen is preserved in a yellowish carbonate concretion that is approximately 70 mm in length and 45 mm in width. The crinoid is preserved as a natural mould and includes the complete theca, articulated arms, and part of the column. The specimen is housed in the Museo Geominero (Madrid, Spain) under the repository number MGM 6754.

**Figure 4:**
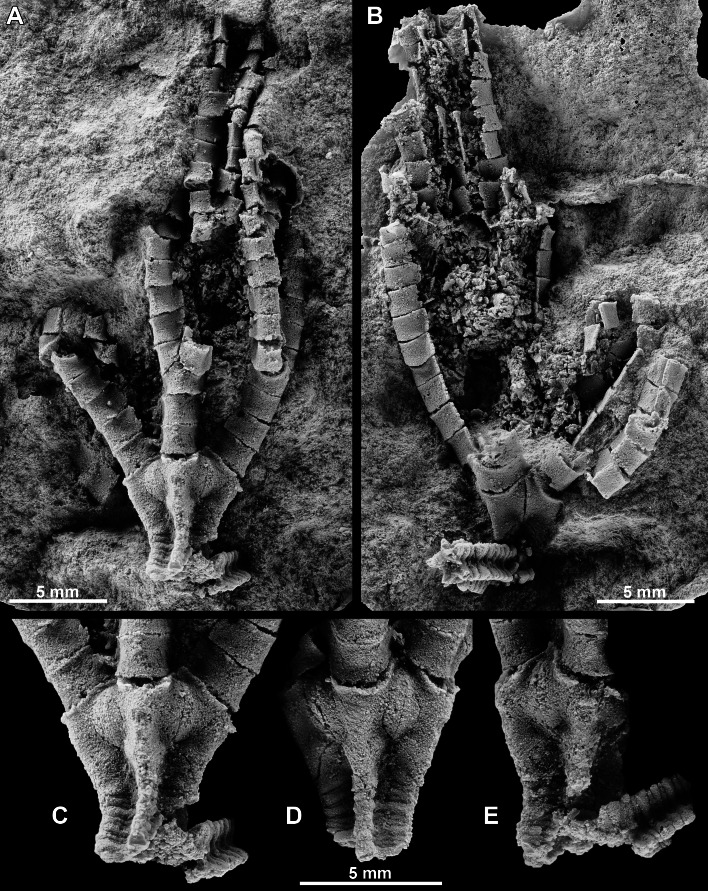
*Iocrinus africanus* sp. nov. (MGM 6754) from the Darriwilian (Middle Ordovician) of Morocco. (A), (B) General morphology including the complete crown showing the E-ray (A) and BC-interray (B), the proximal column, and part of the arms. (C) Detail of the cup showing the E-ray. (D) Detail of the cup showing the A-ray. (E) Detail of the cup showing the D-ray. All images are photographs of latex casts of the specimen whitened with ammonium chloride sublimate.

**Figure 5:**
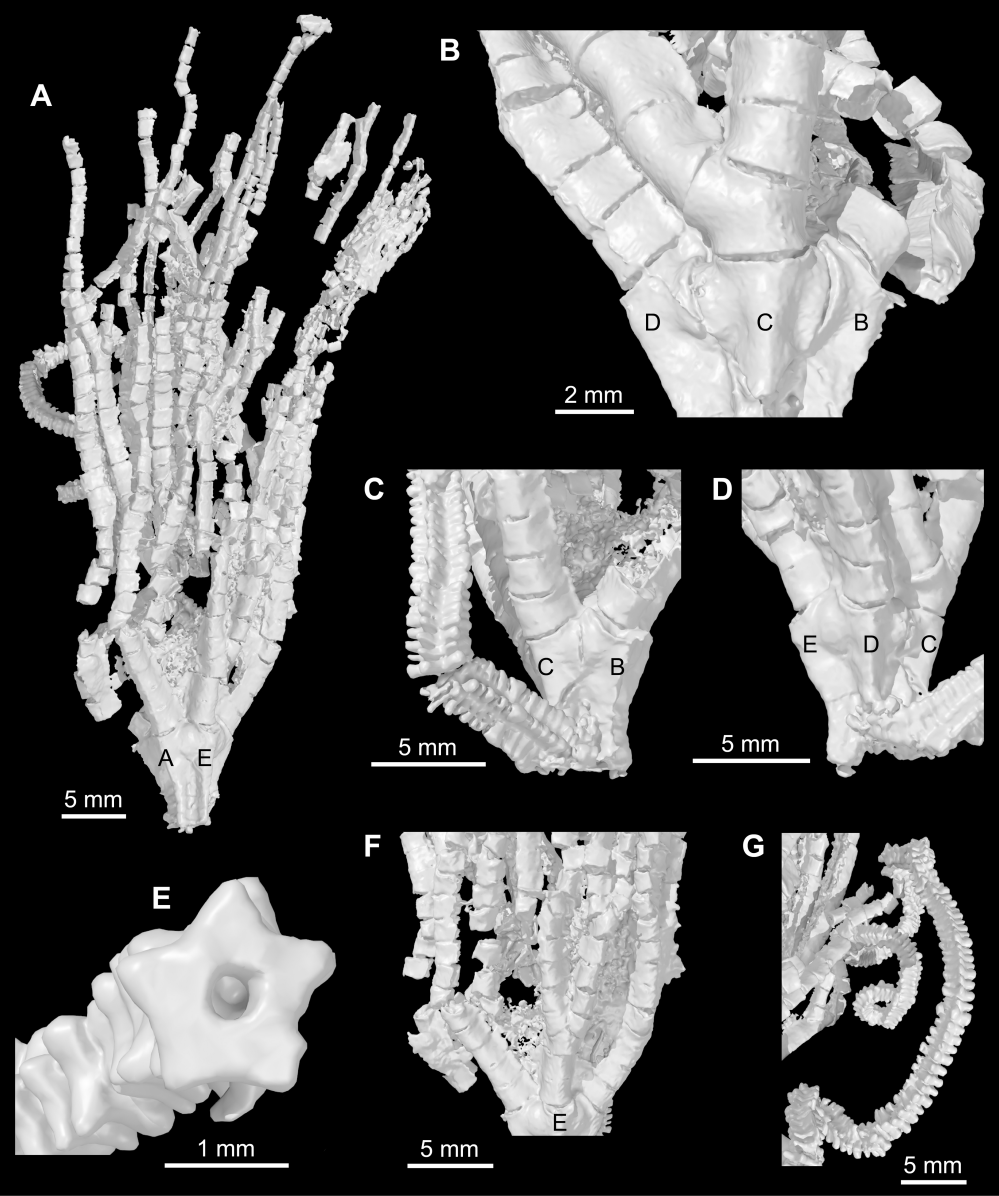
*Iocrinus africanus* sp. nov. (MGM 6754) from the Darriwilian (Middle Ordovician) of Morocco. Digital reconstructions of the specimen. (A) General morphology showing the AE-interray. (B) Detail of the theca showing the C-ray. (C) Detail of the cup showing the BC-interray. (D) Detail of the cup showing the D-ray. (E) Detail of the column showing pentastellate shape and holomeric construction. (F) Detail of the proximal arms showing the E-ray. (G) Column in an open coil. Abbreviations: A–E, ambulacra.

A latex cast of the specimen was prepared to study the morphology of the animal (Fig. 4). In addition, the specimen was imaged using micro-CT and digitally reconstructed to characterize the fossil in three dimensions (Fig. 5). The specimen was scanned on a Nikon XT H 225 cabinet scanner at the Natural History Museum, London with a 0.5 mm thick copper filter, 215 kV voltage, 177 µA current, and 3,142 projections (each with an exposure time of 708 ms). Tomographic reconstruction was performed in Nikon CT Pro software using filtered back projection, giving a tomographic dataset with a voxel size of 37 µm. This dataset was then visualized with the free SPIERS software suite (Sutton et al., 2012); an inverted linear threshold was applied to the dataset, and the pixels that could be unambiguously identified as representing the crinoid were manually assigned to a separate region-of-interest. Isosurfaces were rendered to give an interactive three-dimensional model of the fossil, which was subjected to weak smoothing and island removal to reduce noise. Micro-CT slices, segmented images, and the interactive 3-D model (in VAXML format) are provided at the following DOI http://dx.doi.org/10.5523/bris.uv7qt4c6kpat1befj0937ooml.

### Terminology

The terminology used below follows Moore (1962), Webster (1974), Ubaghs (1978), and Ausich et al. (1999); the classification follows Ausich (1998). Note, the terminology used for the aboral plates differs from that of Ausich, Gil Cid & Domínguez Alonso (2002). In addition, superradial and inferradial are used to designate radially positioned plates where two plates are in the C ray portion of the radial circlet. This usage recognizes the homologies of these plates (essential for phylogenetic analysis) rather than using unique names that obscure homology, such as anibrachial and brachianal as outlined in Ubaghs (1978). The present usage is consistent with many recent studies (e.g., Ausich, 1998; Ausich & Copper, 2010; Ausich et al., 2015; Ausich, Rozhnov & Kammer, 2015), although the Ubaghs (1978) terminology for these plate is also used (e.g., Guensburg, 2010).

### Nomenclatural acts

The electronic version of this article in Portable Document Format (PDF) will represent a published work according to the International Commission on Zoological Nomenclature (ICZN), and hence the new names contained in the electronic version are effectively published under that Code from the electronic edition alone. This published work and the nomenclatural acts it contains have been registered in ZooBank, the online registration system for the ICZN. The ZooBank LSIDs (Life Science Identifiers) can be resolved and the associated information viewed through any standard web browser by appending the LSID to the prefix “http://zoobank.org/”. The LSID for this publication is: http://zoobank.org/References/3F137EAC-ECB5-4FF8-9BA5-8B1A0E1BC986. The online version of this work is archived and available from the following digital repositories: PeerJ, PubMed Central and CLOCKSS.

## Results

## Systematic Paleontology

**Table:** 

Class CRINOIDEA Miller, 1821
Subclass DISPARIDA Moore & Laudon, 1943
Order MYELODACTYLIDA Ausich, 1998
Family IOCRINIDAE Moore & Laudon, 1943
Genus *Iocrinus* Hall, 1866

**Type species**

*Heterocrinus* (*Iocrinus*) *polyxo* Hall, 1866 = *Heterocrinus subcrassus* Meek & Worthen, 1865.

**Diagnosis**

Iocrinid with basal plates visible in lateral view; anal sac with large plicate plates if calcified; variable number of primibrachials; arms branch as many as eight times; fixed interradial plates absent; column holomeric, pentalobate throughout; columnal facets in mesistele petaloid.

*Iocrinus africanus* sp. nov. urn:lsid:zoobank.org:act:D091338E-643F-4D5A-8A08-7D7D190DBC2E.

**Holotype**

MGM 6754, a nearly complete, articulated specimen not retaining the attachment structure and dististele preserved as a mould in a carbonate concretion (Figs. 4 and 5; Data S1, S2, http://dx.doi.org/10.5523/bris.uv7qt4c6kpat1befj0937ooml).

**Type locality and age**

Close to the village of Battou, south Alnif, eastern Anti-Atlas, Morocco (Fig. 2); Taddrist Formation, Darriwilian (Middle Ordovician).

**Etymology**

Named in reference to the African continent.

**Diagnosis**

Basal plate height approximately 37 percent of radial plate height; radial plates 1.25 times higher than wide; single, broad transverse ridge between adjacent radial plates; primibrachials 1.5 times wider than high; three to five primibrachials; four to five secundibrachials; seven to thirteen tertibrachials; three in-line bifurcations per ray; anal sac unplated or very lightly plated (except for the robust column of plates from the C-ray superradial); proximal columnals pentastellate.

### Description

Crown small in size. Aboral cup medium bowl-shaped; smooth plate surfaces; radial and basal plates sharply convex.

Basal circlet 27 percent of aboral cup height; five basal plates, approximately two times wider than high, much smaller than radial plates. Radial circlet 73 percent of aboral cup height; radial plates five, maximum height approximately 1.25 times higher than maximum width; maximum width of radial plate at mid-height, radials narrow sharply proximally, maximum width more than 10 times proximal width; maximum width 1.6 times distal width. Radial facets peneplenary, approximately as deep as wide. A, B, D, E radial plates simple, C radial compound; C inferradial approximately same size as simple radials; C superradial much smaller than C inferradial, wider than high, distal heterotomous division with anal plates to left and C-ray arm to right.

All anal plates above aboral cup; column of 16 stout anal sac plates preserved from the left facet on the C-ray superradial, plates very convex, successive plates with bend yielding a sinuous appearance for this column of plates; each plate higher than wide, otherwise very similar to shape of brachials. Other anal sac plates disarticulated and collapsed within the crown, presumably sac plates were lightly calcified or uncalcified, except for the column of plates from the C superradial.

Arms robust, primaxil varies from third to fifth primibrachial (45553; ABCDE), secundaxil fourth or fifth secundibrachial; where known, tertaxil positioned on the seventh or thirteenth tertibrachial; as many as 16 unbranched quartibrachials on an incomplete branch of the A-ray arm. Brachials strongly convex aborally with flattened lateral, abambulacral extensions, rectangular uniserial, deep ambulcacral groove, more proximal brachials approximately 1.7 times wider than high. Brachial facet with two, merging aboral ligament fossae. Primaxial approximately the same size as non-axillary primibrachials; remaining brachials diminish in size distally.

Column strongly pentastellate, holomeric, heteromorphic, proximal column N3231323; nodals higher than priminternodals, obvious heteromorphic pattern lacking in mesistele, large portion of columnal facets presumably a petaloid articulation (but details not preserved). Preserved column higher than crown height and preserved in an open coil.

### Remarks

Characters differentiating genera within the Iocrinidae are listed in Ausich, Rozhnov & Kammer (2015). The combination of visible basal plates, three to five primibrachials, no fixed interradial plates, pentalobate/pentastellate columnal shape, holomeric column construction, and a petaloid facet clearly align the new crinoid described herein with the genus *Iocrinus*. Another feature that identifies the specimen as belonging to *Iocrinus* is the preservation of the column in an open coil. This is similar to *Iocrinus subcrassus*, which is thought to have had a holdfast that could coil around erect objects (Kelly, 1978; Brett, Deline & McLaughlin, 2008; Meyer & Davis, 2009).

Species-level characters within *Iocrinus* include: the height of the basal plates, the height of the radial plates, radial plate height versus width, presence and character of the transverse ridge between adjacent radial plates, primibrachial shape, number of primibrachials, number of secundibrachials, number of tertibrachials, maximum number of in-line bifurcations in a ray, anal sac plating, and the shape of the proximal columnal (Table 1). *Iocrinus africanus* sp. nov. is distinguished from other *Iocrinus* species based on the shape of the radial plates, the number of tertibrachials, the number of bifurcations in-line per ray, and the lack of or very light plating of most of the anal sac.

**Table 1:** Morphological comparison of *Iocrinus* species.

*Iocrinus* species	Basal plate height	Radial plate height vs. width	Transverse ribbing on between adjacent radial plates	Primibrachial shape	Number of primibrachials	Number of secundibrachials	Number of tertibrachials	Number of arm bifurcations in line	Anal Sac plating robust	Proximal column shape
*Iocrinus crassus*	Approximately 50% of radial plate height	Height approximately equals width	Yes, single, broad	2.0 times wider than high	4 to 5	4 to 6	4 to 8	As many as 7	Unknown	Pentastellate
*Iocrinus llandegleyi*	Approximately 67% of radial plate height	Slightly wider than high	No	2.0 times wider than high	5 to 8	4 to 5	4 to 5	At least 3	Yes	Pentastellate
*Iocrinus pauli*	Approximately 60% of radial plate height	Height approximately equals width	Yes, double, narrow	Less than 2.0 times wider than high	5	5 to 6	5 to 8	4	Yes	Pentalobate
*Iocrinus similis*	Unknown	Height approximately equals width	Unknown	1.5 times wider than high	3 to 4	Unknown	Unknown	Unknown	Unknown	Unknown
*Iocrinus subcrassus* ^a^	Approximately 50% of radial plate height	Height less than width	Yes, single, narrow	2.0 times wider than high	3 to 8	4 to 5	5 to 13	Typically 4 but can be 3 to 8	Yes	Pentalobate
*Iocrinus subcrassus torontoensis*	Approximately 50% of radial plate height	Height approximately equals width	Yes, single, narrow	2.0 times wider than high	5	6 to 7	6 to 11	4	Yes	Pentastellate?
*Iocrinus trentonensis*	Approximately 50% of radial plate height	Height approximately equals width	Yes, single, broad	1.5 times wider than high	4 to 6	6 to 9	>12	4	Yes	Pentalobate?
*Iocrinus whitteryi*	Approximately 67% of radial plate height	Slightly wider than high	No	More than 2.0 times wider than high	7	Unknown	Unknown	Unknown	Yes	Unknown
*Iocrinus africanus* n.sp.	Approximately 37% of radial plate height	1.25 times higher than wide	Yes, single, broad	1.5 times wider than high	3 to 5	4 to 5	7 to 13	3	No	Pentastellate

**Notes.**

Diagnostic table for species of *Iocrinus*

a indicates type species

Donovan et al. (2011) reported the only other putative *Iocrinus* known from Gondwana, *I*. sp. cf. *I. subcrassus* from the Middle Ordovician of Oman. Assuming that this taxon does belong to *Iocrinus*, which cannot be confirmed without further information about the CD-interray and C-ray morphologies, the new Moroccan species differs from the Donovan et al. (2011) specimen as follows. *Iocrinus africanus* sp. nov. has a basal plate height approximately 37 percent of radial plate height; a broad transverse ridge; primibrachials 1.5 times wider than high; four to five secundibrachials; and three in-line bifurcations per ray. In contrast, *I*. sp. cf. *I. subcrassus* has a basal plate height approximately 50 percent of radial plate height; a narrow transverse ridge; primibrachials slightly higher than wide; seven secundibrachials; and as many as seven in-line bifurcations per ray.

Taxonomic assignments within the Iocrinidae have received some attention in the last three decades (Warn, 1982; Guensburg, 1984; Donovan, 1985; Donovan, 1989; Ausich, Rozhnov & Kammer, 2015); with the new species described herein, a total of eight species and one subspecies are currently recognized for *Iocrinus* (Webster & Webster, 2014). These include the Laurentian species: *I. crassus* (Meek & Worthen, 1865); *I. similis* (Billings, 1865); *I. subcrassus* (Meek & Worthen, 1865); *I. subcrassus torontoensis* (Fritz, 1925); and *I. trentonensis* (Walcott, 1884); and the Avalonian species: *I. llandegleyi* (Botting, 2003); *I. pauli* (Donovan & Gale, 1989); and *I. whitteryi* (Ramsbottom, 1961) (Table 2). Additional *Iocrinus* identifications left in open nomenclature are known from Avalonia, Laurentia, and Gondwana (for the previous potential Gondwanan occurrence, see Donovan et al., 2011). *Iocrinus africanus* sp. nov. is Darriwilian in age, and thus it is among the oldest members of the genus (Table 2). In terms of morphology, it is equally dissimilar to species from both Laurentia and Avalonia. The occurrence of *I. africanus* sp. nov. in Morocco confirms the presence of *Iocrinus* in Gondwana and demonstrates that *Iocrinus*, together with *Merocrinus*, is the most geographically widespread Ordovician crinoid genus.

**Table 2:** Stratigraphic and geographical distribution of species of *Iocrinus* and *Merocrinus*.

Genus	Species	Formation	Age	Location	Country	Paleo-continent
*IOCRINUS*						
	*Iocrinus* sp. cf*. I. subcrassus*	Amdeh Formation	late Dapingian or early Darriwilian	Muscat	Oman	Gondwana
	*Iocrinus llandegleyi*	Builth Volcanic Group	Darriwillian	Wales	UK	Avalonia
	*Iocrinus pauli*	Camnant Mudstone	Darriwillian	Wales	UK	Avalonia
	*Iocrinus pauli*	Didmograptus bifidus Beds	Darriwillian	England	UK	Avalonia
	*Iocrinus* sp. cf. *pauli*	Llandeilo Flags	Darriwillian	Wales	UK	Avalonia
	*Iocrinus* cf. *whitteryi*	volcanic sandstones	Darriwillian	England	UK	Avalonia
	*Iocrinus whitteryi*	Chirbury Formation	Sandbian	England	UK	Avalonia
	*Iocrinus* cf. *subcrassus*	Whittery Beds	Sandbian	England	UK	Avalonia
	*Iocrinus subcrassus*	Arnheim Formation	Katian	Southwestern Ohio Region	USA	Laurentia
	*Iocrinus subcrassus*	Lorraine Shale	Katian	New York	USA	Laurentia
	*Iocrinus* cf*. subcrassus*	Lorraine Shale	Katian	New York	USA	Laurentia
	*Iocrinus subcrassus*	Cobourg Limestone	Katian	Ontario	Canada	Laurentia
	*Iocrinus subcrassus*	Georgian Bay Formation	Katian	Ontario	Canada	Laurentia
	*Iocrinus subcrassus torontoensis*	Dundas Formation	Katian	Ontario	Canada	Laurentia
	*Iocrinus similis*	Cobourg Limestone	Katian	Ontario	Canada	Laurentia
	*Iocrinus subcrassus*	Correyville Formation	Katian	Southwestern Ohio Region	USA	Laurentia
	*Iocrinus* sp.	Fort Atkinson Formation	Katian	Iowa and Illinois	USA	Laurentia
	*Iocrinus subcrassus*	Fairview Formation	Katian	Southwestern Ohio Region	USA	Laurentia
	*Iocrinus* sp.	Kope Formation	Katian	Southwestern Ohio Region	USA	Laurentia
	*Iocrinus subcrassus*	Liberty Formation	Katian	Southwestern Ohio Region	USA	Laurentia
	*Iocrinus crassus*	Maquoketa Shale	Katian	Illinois	USA	Laurentia
	*Iocrinus trentonensis*	Rust Formation	Katian	New York	USA	Laurentia
	*Iocrinus trentonensis*	Trenton Limestone	Katian	New York	USA	Laurentia
	*Iocrinus subcrassus*	Waynesville Formation	Katian	Southwestern Ohio Region	USA	Laurentia
*MEROCRINUS*						
	*Merocrinus millanae*	Guindo Shales	Darriwilian	Embalse de Fresneda	Spain	Gondwana
	*Merocrinus salopioe*	Meadowtown Beds	Darriwilian		England	Avalonia
	*Merocrinus britonensis*	Mifflin Formation	Sandbian	Illinois	US	Laurentia
	*Merocrinus britonensis*	Platteville Group	Sandbian	Illinois, Iowa, Wisconsin, Minn	US	Laurentia
	*Merocrinus impressus*	Bromide Formation (Pooleville Mbr.)	Sandbian	Oklahoma	US	Laurentia
	*Merocrinus impressus*	?	?	?	Sweden	Baltica
	*Merocrinus curtus*	Kope Formation	Katian	Southwestern Ohio Region	US	Laurentia
	*Merocrinus curtus*	Rust Formation	Katian	New York	US	Laurentia
	*Merocrinus retractilis*	Rust Formation	Katian	New York	US	Laurentia
	*Merocrinus* sp.	Wisf Formation (Sinsinewa Mbr.)	Katian	Illinois and Iowa	US	Laurentia
	*Merocrinus corroboratus*	Trenton Limestone	Katian	New York	US	Laurentia
	*Merocrinus typus*	Trenton Limestone	Katian	New York	US	Laurentia

The use of micro-CT was essential for describing the morphology of *Iocrinus africanus* sp. nov. in full. The posterior interray is buried below the surface of the concretion and is hence not visible in the latex casts (Fig. 4); however, the posterior interray and the C-ray can be clearly seen in the micro-CT scans (Fig. 5; Data S1, S2, http://dx.doi.org/10.5523/bris.uv7qt4c6kpat1befj0937ooml). Without an understanding of these characters, it would not have been possible to confidently assign the specimen to the genus *Iocrinus*.

## Paleobiogeographical Implications

**Figure 6:**
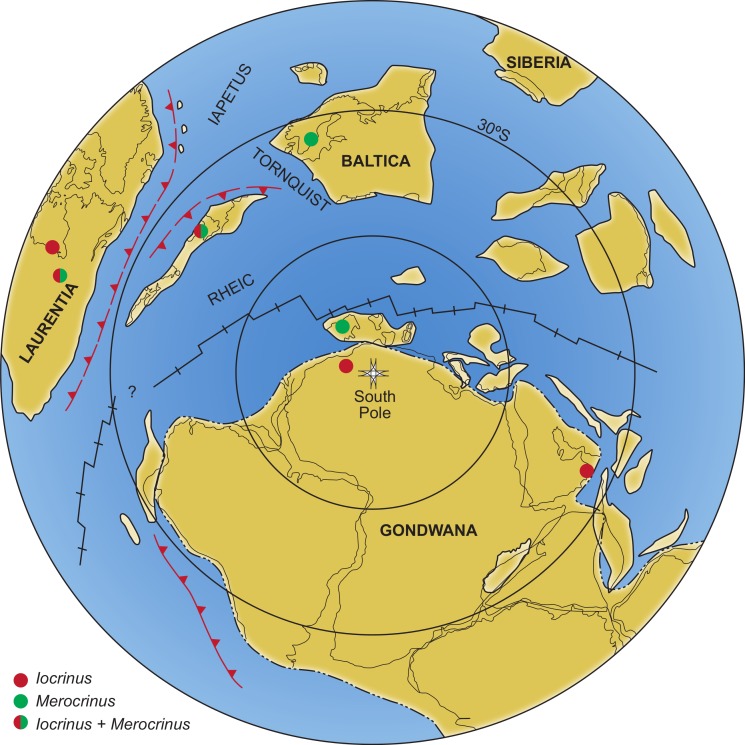
Distribution of the major paleocontinents during the Middle Ordovician, showing the known geographical distribution of *Iocrinus* and *Merocrinus*. Locality markers indicate the presence of a taxon on a palaeocontinent; multiple localities are not noted on a single palaeocontinent. Modified from Cocks & Torsvik (2006).

The Middle to Late Ordovician was characterized by high degrees of endemism in crinoids (Paul, 1976; Lefebvre et al., 2013), and *Iocrinus* and *Merocrinus* are the only geographically widespread genera from this period (Fig. 6). Both genera first appeared in Gondwana and/or Avalonia during the Darriwillian. *Merocrinus* first occurred in Laurentia during the Sandbian, and *Iocrinus* first occurred in Laurentia during the Katian (however, Sprinkle, Guensburg & Gahn, 2008 noted the occurrence of older, undescribed iocrinids and a merocrinid-like cladid? from faunas in North America). Based on presently described taxa, the known geographical distribution of these genera indicates that their migration to Laurentia was asynchronous. *Iocrinus* is a disparid crinoid, and disparids are usually recognized as having a more widespread geographic distribution and temporal range than other clades (Kammer, Baumiller & Ausich, 1998). *Merocrinus* is generally considered to be a cladid (but see Sprinkle & Guensburg, 2013), which in general are not as cosmopolitan as disparids, at least later during the Paleozoic. Unfortunately, there is not currently enough known about the life history of Paleozoic crinoids to propose any explanation for the cosmopolitan nature of *Iocrinus* and *Merocrinus* during the Ordovician.

## Additional Information and Declarations

### Data Availability

The following information was supplied regarding data availability: Palaeogeographic implications of a new iocrinid crinoid (Disparida) from the Ordovician (Darriwillian) of Morocco DOI http://dx.doi.org/10.5523/bris.uv7qt4c6kpat1befj0937ooml.

### New Species Registration

The following information was supplied regarding the registration of a newly described species: Iocrinus africanus Zamora, Rahman & Ausich: urn:lsid:zoobank.org:act:D091338E-643F-4D5A-8A08-7D7D190DBC2E Publication LSID: http://zoobank.org/References/3F137EAC-ECB5-4FF8-9BA5-8B1A0E1BC986.
